# Flow evaluation software for four-dimensional flow MRI: a reliability and validation study

**DOI:** 10.1007/s11547-023-01697-4

**Published:** 2023-08-24

**Authors:** Barbara Elisabeth Ursula Burkhardt, Christian Johannes Kellenberger, Fraser Maurice Callaghan, Emanuela Regina Valsangiacomo Buechel, Julia Geiger

**Affiliations:** 1 Paediatric Cardiology, Pediatric Heart Center, Department of Surgery, University Children’s Hospital Zürich, Steinwiesstrasse 75, 8032 Zurich, Switzerland; 2 Department of Diagnostic Imaging, University Children’s Hospital Zürich, Zurich, Switzerland; 3 Children’s Research Center, University Children’s Hospital Zürich, Zurich, Switzerland

**Keywords:** 4D flow MRI, Phase-contrast magnetic resonance imaging, Cardiac magnetic resonance, Haemodynamics, Flow quantification, Congenital heart disease

## Abstract

**Purpose:**

Four-dimensional time-resolved phase-contrast cardiovascular magnetic resonance imaging (4D flow MRI) enables blood flow quantification in multiple vessels, which is crucial for patients with congenital heart disease (CHD). We investigated net flow volumes in the ascending aorta and pulmonary arteries by four different postprocessing software packages for 4D flow MRI in comparison with 2D cine phase-contrast measurements (2D PC).

**Material and methods:**

4D flow and 2D PC datasets of 47 patients with biventricular CHD (median age 16, range 0.6–52 years) were acquired at 1.5 T. Net flow volumes in the ascending aorta, the main, right, and left pulmonary arteries were measured using four different postprocessing software applications and compared to offset-corrected 2D PC data. Reliability of 4D flow postprocessing software was assessed by Bland–Altman analysis and intraclass correlation coefficient (ICC). Linear regression of internal flow controls was calculated. Interobserver reproducibility was evaluated in 25 patients.

**Results:**

Correlation and agreement of flow volumes were very good for all software compared to 2D PC (ICC ≥ 0.94; bias ≤ 5%). Internal controls were excellent for 2D PC (*r* ≥ 0.95, *p* < 0.001) and 4D flow (*r* ≥ 0.94, *p* < 0.001) without significant difference of correlation coefficients between methods. Interobserver reliability was good for all vendors (ICC ≥ 0.94, agreement bias < 8%).

**Conclusion:**

Haemodynamic information from 4D flow in the large thoracic arteries assessed by four commercially available postprocessing applications matches routinely performed 2D PC values. Therefore, we consider 4D flow MRI-derived data ready for clinical use in patients with CHD.

**Supplementary Information:**

The online version contains supplementary material available at 10.1007/s11547-023-01697-4.

## Introduction

The assessment of haemodynamics is an essential part of cardiovascular magnetic resonance imaging (MRI) for diagnosing and monitoring cardiovascular disease, especially in children and adults with congenital heart disease (CHD). In recent years, four-dimensional (4D) flow MRI referring to an ECG-gated, time-resolved three-dimensional (3D) phase-contrast (PC) sequence with flow-encoding in all three spatial directions has become available as a diagnostic tool to non-invasively quantify blood flow [1]. The main advantages of a 4D flow sequence are that its acquisition is simpler for the operator than a two-dimensional (2D) cine PC sequence and that blood flow can retrospectively be evaluated in any desired plane within the acquired volume. This is particularly advantageous in patients with CHD, in whom often multiple flow measurements need to be obtained for assessing shunts, blood flow distribution or regurgitation volumes [2–7]. Clinical examples include the calculation of shunts between the systemic and the pulmonary circulations in patients with septal defects or aberrant pulmonary venous connections [2] or the determination of pulmonary blood flow distribution in patients after pulmonary artery surgery as in tetralogy of Fallot [4] or in single ventricle defects with cavopulmonary anastomoses [6]. In addition to flow volumes, parameters such as flow velocities, three-dimensional flow patterns (helicity, vorticity), vascular wall shear stress or kinetic energy may be obtained from 4D flow MRI datasets [8].

Until lately, the main challenges and limitations for widespread clinical use of 4D flow MRI have been its complex evaluation and long acquisition and postprocessing times. However, recent improvements in scanning acceleration techniques and the advent of commercial postprocessing software have facilitated routine clinical application of 4D flow MRI. Most MR vendors offer a dedicated 4D flow sequence with up-to-date acceleration methods combined with advanced cardiovascular postprocessing packages, both with scanning and assessment times reasonable for clinical use [9–11]. In addition, there are several commercial or custom-made software solutions for analysing four-dimensional flow datasets [1] with differing capabilities and features. They all include the possibility to measure antegrade and retrograde through-plane flow for calculation of net flow volume in vessels. Each software package has its own method for correcting residual phase errors due to gradient non-linearity, Maxwell fields and eddy currents that were not completely accounted for during acquisition [1, 11–13]. Implementation and performance of such phase-offset correction methods may vary across MRI systems and postprocessing applications [1].

The aim of this work was to compare the clinical applicability, reliability, and validity of different commercially available postprocessing software packages for flow volume quantification on 4D flow MRI data in comparison to 2D PC measurements employing phase-offset corrections with static gel phantoms as a reference.

## Material and methods

### Patient population

Consecutive patients who had undergone cardiac MRI between March and July 2018 at our tertiary referral cardiac centre were eligible for retrospective review. All patients with biventricular physiology who were scanned using both sequences (2D PC sequences through the ascending aorta (AAO), main pulmonary artery (MPA), right (RPA) and left (LPA) pulmonary arteries as well as a 4D flow acquisition covering the chest) were included in the study. Patients with Fontan circulation or patients without written general consent for research use of health-related data were not considered, the former because Fontan physiology lacks a ventriculo-pulmonary artery with pulsatile flow.

### Image acquisition

All examinations were performed on a 1.5 Tesla scanner (Discovery MR450, GE Healthcare, Waukesha, WI, USA) with a 32-channel phased-array cardiac coil covering the chest. Following static 2D steady-state free precession localisers in three orthogonal planes, 2D cine steady-state free precession sequences were acquired in standard planes aligned to the heart axes for assessing cardiac morphology and function. Contrast-enhanced 3D spoiled gradient-echo angiography (contrast medium gadoteric acid; Dotarem, Guerbet AG; Zürich, Switzerland at a dose of 0.1–0.2 mmol/kg body weight; 10 mmol maximum dose) was acquired for assessing vascular morphology.

The 2D PC planes were prescribed during the cardiac MRI examination perpendicular to the course of the vessels: at the level of the pulmonary arteries for the AAO, between the pulmonary valve and pulmonary bifurcation for the MPA, posterior to the ascending aorta for the RPA, and between the pulmonary bifurcation and the origin of the anterior segmental arterial branch for the LPA. The field of view was adjusted to the size of the patient. Standard velocity encoding was 200 cm/s. When aliasing was detected, the measurement was repeated with a higher encoding velocity. The 2D PC measurements were acquired with breath holding at expiration in 32/47 (68%) patients (with 1 excitation) and during quiet breathing in 15/47 (32%) patients (with 2 or 3 excitations). At the end of the examination, all 2D PC measurements were repeated on a static gel phantom and with identical technical parameters for phase-offset correction.

The 4D flow sequence was acquired in free breathing immediately after contrast-enhanced MR angiography in transverse orientation covering the aortic arch cranially and the apex of the heart caudally, with retrospective ECG gating. We used a short echo time (TE) and repetition time (TR) radiofrequency-spoiled gradient-echo sequence accelerated by kt-ARC, a spatiotemporal-correlation-based auto calibrating parallel imaging method allowing for a median acquisition time of 9 min (range 6–14 min) in these patients. Radial golden angle view order in ky-kz and variable density number of excitations (NEX) scheme was used for motion robust imaging with little loss in scan efficiency. Views per segment and degree of acceleration were automatically set by the sequence depending on the heart rate, desired number of temporal phases per cardiac cycle, and spatial resolution. We aimed at 20 or more acquired temporal phases per cardiac cycle and an isotropic acquired spatial resolution between 1.6 mm^3^ in infants and 2.4 mm^3^ in large adults. A standard velocity encoding of 160 cm/s was used. This was increased if velocities exceeding 200 cm/s were seen on the 2D PC images. The acquisition parameters are detailed in Supplemental Table 1.

### Flow evaluation

Net flow volumes were measured in all AAO, MPA, RPA and LPA. Pulmonary-to-systemic blood flow ratio (Q*p*/Q*s*) was calculated as the ratio of flow volumes in MPA and AAO. Differential pulmonary blood flow was expressed as percentage of the flow volume to the right lung (%RPA) and calculated from flow measurements in RPA and LPA [net flow volumes (RPA/(RPA + LPA) × 100].

The 2D PC images were analysed with Qflow version 8.1 (Medis Suite 3.0, MEDIS Medical Imaging Systems, Leiden, The Netherlands) by the cardiologist or radiologist performing the clinical MRI study. Semi-automatic contour detection and phase-offset correction with data from the corresponding static gel phantom scan was applied.

**Fig. 1:**
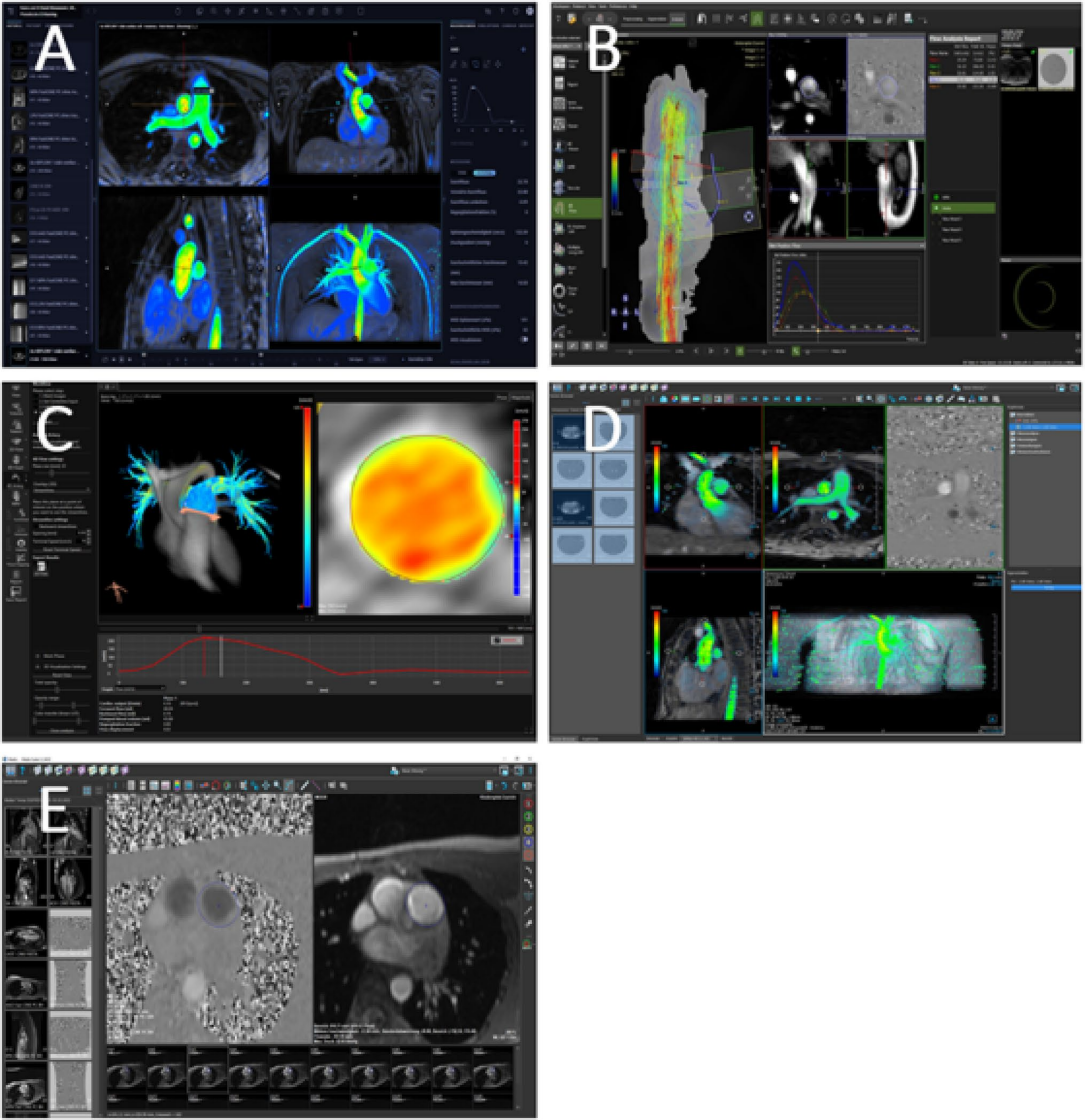
Examples of software user interfaces for the 4D flow software programmes at the level of plane definition (panels **A**–**D**. **A**: Arterys; **B**: Circle; **C**: Caas; **D**: Medis) and for 2D flow placement of regions of interest (panel **E**)

The 4D flow images were processed by two experienced paediatric radiologists and one paediatric cardiologist (between 4 and > 10 years of experience) with 4 different software packages: A) Arterys (Cardio AI^MR^, Arterys Inc., San Francisco, CA, USA), which is a cloud-based image reconstruction platform [11, 13] and with locally installed applications, B) Circle (cvi^42^, version 5.6, Circle Cardiovascular Imaging Inc., Calgary, Canada), C) Caas (Caas MR Solutions, Version 5.0, Pie Medical Imaging, Maastricht, The Netherlands), and D) Medis (Qflow 4D, Version 1.1, Medis Suite MR 3.2, MEDIS Medical Imaging Systems, Leiden, The Netherlands) (Fig. 1). For assessing interobserver agreement, 25 cases were processed by two independent readers for each software. Background velocity correction was performed by the phase-offset correction methods provided by the individual software. In addition, one flow assessment without phase-offset correction was obtained with software A. The measurement planes were placed as described for 2D PC, and vessel contours were traced semi-automatically to include the entire flow volume in all phases of the cardiac cycle. Contour placement and shape were double-checked and manually corrected as needed on magnitude as well as on velocity images. In case of aliasing, phase unwrapping was used for the entire 4D volume in software B. In the other software programmes, phase unwrapping features were not available, and measurement planes had to be adjusted to locations free from aliasing. Postprocessing time was measured in 16 patients during the second half of the study, allowing for some practice with each software during analysis of the first patients.

### Comparisons for 4D flow software and statistical analysis

Continuous data with normal distribution are given as mean ± standard deviation (SD) and data without normal distribution as median and interquartile range (IQR). Frequencies are given as fraction and percentage. Normal distribution of the data was assessed with Shapiro–Wilk test. Reliability and validity of 4D flow measurements obtained with software A–D were evaluated by comparing net flow volumes, haemodynamic measures (Qp/Qs and %RPA) and internal controls to 4D flow measurements without phase-offset correction and to phase-offset-corrected 2D PC data. Net flow volumes of all vessels were compared with the Wilcoxon test. Agreement for assessing flow volumes was evaluated with intraclass correlation (ICC) for absolute agreement of single measures and Bland–Altman analysis. Absolute percentage error was calculated. For checking the consistency of the flow measurements within a dataset, internal controls based on the “conservation of mass” principle were performed: in the absence of a shunt, net flow volume in the AAO should be the same as in the MPA and the sum of net flow volumes in RPA and LPA should be the same as in the MPA. Internal controls were performed with Pearson correlation and Bland–Altman analysis. Interreader agreement was assessed with ICC and Bland–Altman analysis. Correlation coefficients were compared using Fisher r to z transformation.

The statistical analysis was performed with MedCalc Statistical Software version 19.0.5 (MedCalc Software Ltd., Ostend, Belgium). A *P*-value < 0.05 was considered statistically significant.

## Results

### 4D versus 2D flow volumes

This study included MRI examinations from 47 patients with ages ranging from 8 months to 52 years. Patient characteristics and cardiovascular diagnoses/indications for MRI are detailed in Supplemental Table 2. Net flow volumes per cardiac cycle were measured in a total of 188 thoracic arteries (47 AAO, 47 MPA, 47 RPA and 47 LPA). The net flow volumes obtained with software A–D from 4D flow data were significantly lower than those from 2D PC and 4D flow data without phase-offset correction with median differences ranging from − 1.3 to − 3.6 ml per heart beat (Table 1).

**Table 1:** Net flow volume per cardiac cycle in 188 vessels assessed by 2D PC and 4D flow with different postprocessing software programmes

	Net flow volume [ml]	Comparison to 2D PC	
	Median (IQR)	*P*-value *	Median difference, ml
Software A	39.4 (28.6–61.2)	< 0.001	− 3.0
Software B	42.3 (29.2–61.1)	< 0.001	− 1.9
Software C	42.1 (30.2–62.5)	0.005	− 1.3
Software D	40.8 (29.3–2.9)	< 0.001	− 2.1
4D flow without offset correction (Software A)	46.0 (27.9–65.4)	0.335	0.8
2D PC	43.0 (30.3–62.0)		

	Comparison to 4D flow without phase-offset correction	
	*P*-value*	Median difference, ml
Software A	< 0.001	− 3.6
Software B	0.007	− 2.0
Software C	0.034	− 1.6
Software D	< 0.001	− 2.5

^*^Wilcoxon test. Software A: Arterys; software B: Circle; software C: Caas; software D: Medis

### Agreement of 4D flow measurements:

**Fig. 2:**
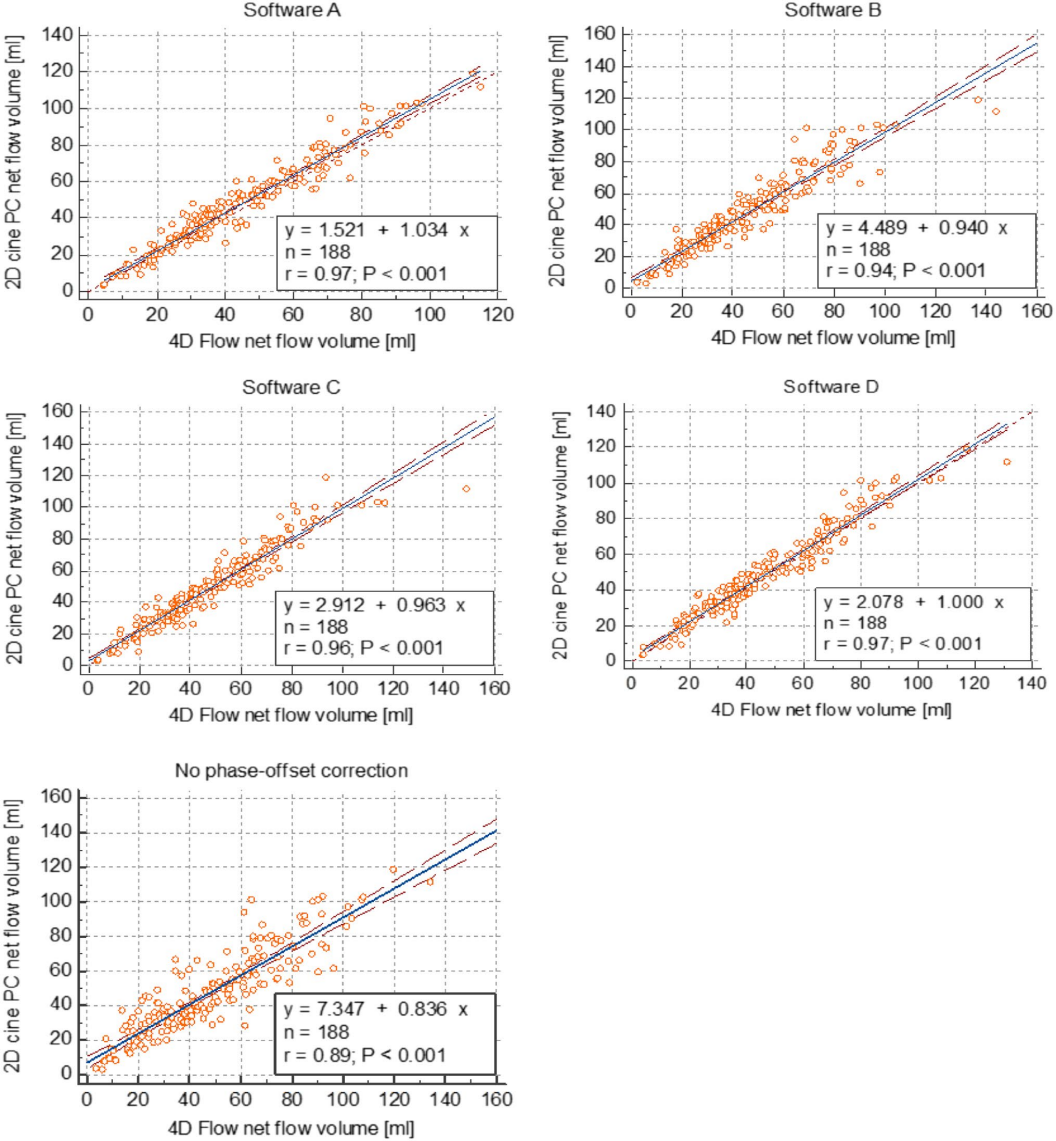
Scatter plots of net flow volume assessment in 188 thoracic arteries by 4D flow compared to 2D PC. Software **A**–**D** employing phase-offset correction showed higher linear correlation (*r* 0.94–0.97) than without phase-offset correction (*r* 0.89). Software **A**: Arterys; software **B**: Circle; software **C**: Caas; software **D**: Medis

The correlation and Bland–Altman analyses of net flow volume, pulmonary-to-systemic flow ratio and differential pulmonary blood flow assessments by 4D flow versus 2D PC are detailed in Table 2. Correlation of flow volumes was significantly better for software A–D employing phase-offset correction (ICC 0.94–0.97) than without phase-offset correction (ICC 0.89, *p* ≤ 0.002) (Fig. 2). The correlation of pulmonary-to-systemic flow ratios (Q*p*/Q*s*) and right-to-left pulmonary flow ratios was also higher when phase-offset correction was used. Pulmonary-to-systemic flow ratio showed high correlation with software A–D (ICC 0.85–0.91) and lower correlation without phase-offset correction (ICC 0.55). Right-to-left pulmonary flow ratios showed overall lower correlation (software A–D ICC 0.33–0.47; without phase-offset correction ICC 0.15). Assessment of both ratios had an absolute error below 10% for each software.

**Table 2:** Agreement between haemodynamic data obtained with different 4D flow postprocessing software programmes and phase-offset-corrected 2D PC data in 47 patients

	Intraclass correlation		Bland–Altman analysis		Absolute percentage error	
	ICC	95% CI	Bias	Range of agreement	Median (%)	95% CI
*Net flow volume*						
Software A	0.96*	0.92–0.98	− 3.1 ml (− 4.4%)	24 ml (61%)	10.2	8.9–11.6%
Software B	0.94*	0.92–0.96	− 1.7 ml (− 2.2%)	32 ml (72%)	11.3	10.3–12.8%
Software C	0.96*	0.94–0.97	− 1.2 ml (− 0.8%)	28 ml (70%)	8.9	7.7–10.6%
Software D	0.97*	0.95–0.98	− 2.1 ml (− 3.1%)	23 ml (63%)	10.3	8.6–11.5%
*Pulmonary-to-systemic flow ratio (Qp / Qs)*						
Software A	0.89*	0.82–0.94	0.02 (2.6%)	0.48 (44%)	8.3	7.4–10.3%
Software B	0.88*	0.74–0.94	0.08 (6.7%)	0.47 (45%)	8.3	4.9–11.3%
Software C	0.85*	0.68–0.93	0.07 (7.8%)	0.57 (57%)	9.6	6.8–12.1%
Software D	0.91*	0.83–0.95	0.04 (4.6%)	0.44 (46%)	6.5	4.2–8.9%
*Differential blood flow (RPA flow percentage)*						
Software A	0.33	0.06–0.56	− 1.0%	27%	7.0	5.1–8.9%
Software B	0.35	0.08–0.58	1.4%	29%	7.8	4.7–10.8%
Software C	0.39	0.12–0.60	− 1.6%	29%	6.5	4.0–11.4%
Software D	0.47	0.22–0.67	1.5%	25%	6.0	4.1–11.7%

*ICC* Intraclass correlation coefficient, *CI* Confidence interval, *SD* Standard deviation

^*^Significantly higher ICC than for 4D flow without phase-offset correction. Software A: Arterys; software B: Circle; software C: Caas; software D: Medis

### Internal controls of flow consistency

The internal controls of flow consistency (Table 3) were best for 2D PC, followed by phase-offset-corrected 4D flow data which were all better than 4D flow data without phase-offset correction (without offset correction: AAO versus MPA *r* = 0.85 (0.73–0.92), bias 8 ml (13.1%); MPA versus RPA + LPA *r* = 0.86 (0.76–0.92), bias 2.4 ml (4.5%)). Internal controls were not significantly different between software A–D with correlation coefficients ranging from 0.94 to 0.98 for the flow volumes in AAO versus MPA, and from 0.95 to 0.97 for the flow volume in MPA versus sum of flow volumes in RPA and LPA.

**Table 3:** Internal controls of flow consistency for 2D PC data and 4D flow data in 47 patients

	Pearson correlation		Bland–Altman analysis	
	*r*	95% CI	Bias	Range of agreement
*Q*_*P*_* = Q*_*S*_* (MPA = AAO)*^*a*^				
2D PC	0.98	0.95–0.99	1.4 ml (1.8%)	19.6 ml (30.2%)
Software A	0.94*	0.89–0.97	3.1 ml (5.1%)	27.8 ml (49.8%)
Software B	0.96*	0.93–0.98	5.7 ml (8.5%)	27.4 ml (43.7%)
Software C	0.94*	0.89–0.97	6.8 ml (8.8%)	38.9 ml (44.4%)
Software D	0.98*	0.96–99	5.0 ml (7.8%)	18.9 ml (30.4%)
*MPA = RPA + LPA*				
2D PC	0.95	0.91–0.97	0.1 ml (0.9%)	30.8 ml (47.0%)
Software A	0.96*	0.92–0.98	2.0 ml (3.0%)	25.8 ml (44.9%)
Software B	0.95*	0.90–0.97	− 1.1 ml (− 1.9%)	33.6 ml (44.6%)
Software C	0.97*	0.95–0.98	1.3 ml (2.1%)	24.1 ml (36.1%)
Software D	0.97*	0.95–0.98	0.8 ml (0.1%)	23.0 ml (49.1%)

^a^in 41 patients without shunt, * significantly higher correlation coefficient r than for 4D flow without phase-offset correction. Software A: Arterys; software B: Circle; software C: Caas; software D: Medis

### Interobserver agreement for 4D flow analysis software programmes

**Fig. 3:**
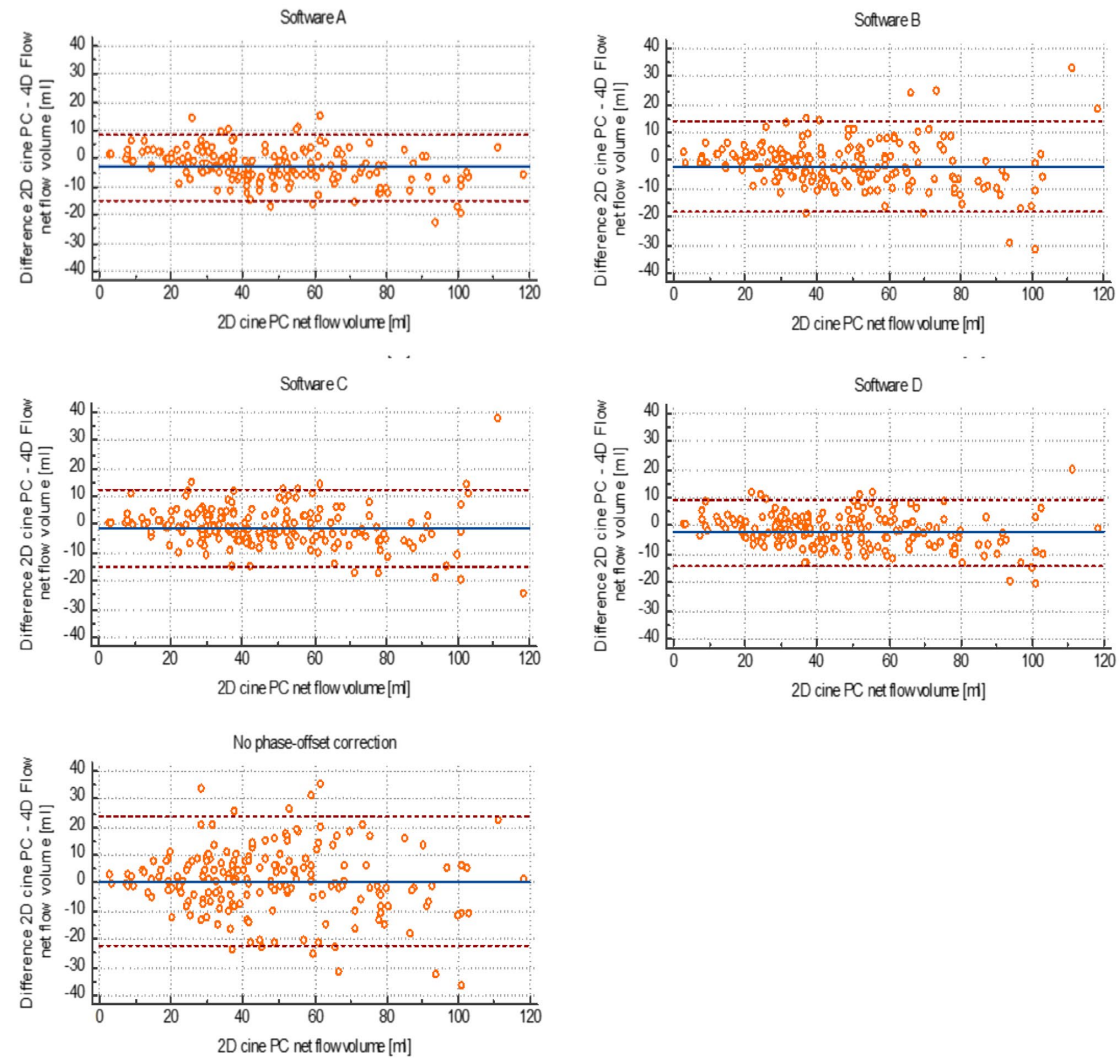
Agreement of net flow volume assessment in 188 thoracic arteries by 4D flow compared to 2D PC. Bland Altman plots show better agreement for software **A**–**D** employing phase-offset correction than without phase-offset correction. Limits of agreement (dotted lines) are narrower when phase-offset correction is used. Systematic differences (solid blue lines in the middle) are small with bias < 4 ml (5%) for all methods, but per cent median error is less with phase-offset correction (9–11%) than without (17%). Complete data of Bland Altman analysis is given in Table 4. Software **A**: Arterys; software **B**: Circle; software **C**: Caas; software **D**: Medis

Interobserver agreement was good for software A, C and D, with significantly higher correlation coefficients and narrower ranges of agreement than for software B (Table 4; Fig. 3).

**Table 4:** Interobserver agreement for measuring net flow volumes by 4D flow software in 96 vessels of 24 patients

	Intraclass correlation		Bland–Altman analysis	
	ICC	95% CI	Bias	Range of agreement
Software A	0.97*	0.87–0.99	−3.6 ml ( −7.7%)	16.8 ml (34.5%)
Software B	0.94	0.89–0.97	3.4 ml (6.1%)	28.3 ml (67.9%)
Software C	0.99*	0.98–0.99	−0.8 ml ( −2.5%)	14.4 ml (32.3%)
Software D	0.99*	0.98–0.99	1.0 ml (2.3%)	14.3 ml (46.7%)

*ICC* Intraclass correlation coefficient, *CI* Confidence interval

*significantly higher ICC than for software B. Software A: Arterys; software B: Circle; software C: Caas; software D: Medis

### Postprocessing time

Postprocessing time for the 4 thoracic arteries per patient from 4D flow datasets was shortest for software A (median 13 min, IQR 12–15 min), followed by software D (median 14 min, IQR 11–17 min, software B (median 14 min, IQR 13–17 min) and software C (median 20 min, IQR 17–23 min).

## Discussion

With the advent of faster 4D flow sequences and user friendly postprocessing software, the clinical application of 4D flow MRI for flow volume measurement in thoracic vessels has become feasible. In this study, we validate 4D flow volume assessment with four postprocessing software packages against phase-offset-corrected 2D PC flow volume measurements in 47 children and adults with congenital heart disease.

Overall, the net flow volumes in the aorta and pulmonary arteries assessed by 4D flow MRI showed good agreement with 2D PC acquisitions, as long as residual phase-offset errors were accounted for by the postprocessing software.

In the clinical setting, 2D PC measurements have been routinely used for many years for calculation of cardiac output, shunt flow, and valve regurgitation. Flow measurements have to deliver reliable and accurate results in order be clinically acceptable and valuable. Therefore, several previous studies have investigated the impact of velocity offset errors on flow measurements [14–18].

Instead of using time-consuming phantom correction measurements, interpolation-based offset corrections serve as an alternative to correct the in vivo data during postprocessing as presented in a multi-vendor and multi-centre study [19]. Another option is field monitoring data in order to analyse and correct for spatiotemporal background velocity offsets induced by eddy currents [18].

We have previously shown that different methods of background phase correction influence flow volume measurements [20]. In this study, we tested different postprocessing platforms which use different ways of identifying and fitting static tissue for background phase correction. Arterys 4D flow module uses a piecewise linear polynomial model with Gaussian smoothing, with semi-automatic static tissue detection based on artificial intelligence. In cases of artefacts, thresholds were adjusted manually. Circle uses a polynomial fit to the velocity values of static tissue and subtracts this from all voxels, based on a previously described approach by Lankhaar et al. for 2D PC [17], and we used the semi-automatic static tissue detection with manual threshold adjustment provided by the programme. The CAAS application corrects eddy currents by fitting a first-order surface through the time average velocities of the stationary tissue pixels of each frame and subtracting it from the original velocity images [16, 17], without modifications by the user. Medis applies second order fitting on the velocity of the automatically determined static tissue, which consists of the 25% of the volume with the lowest standard deviation in velocity.

For 2D PC analysis, we used Medis, which has been evaluated by others in comparison with other analysis software programmes for 2D PC flow volumes in the past. Minderhoud et al. [21] found important phase-offset errors in 2D PC acquisitions that needed phantom correction, which however resulted in the same mean net flow for all tested software programmes (Medis QFlow, Circle cvi 42, and MASS). A comparison of 2D PC analysis with Circle, Argus, and Medis resulted in only small differences between the three, such that they may be used interchangeably [22]. An animal study in swine showed the same stroke volumes in the ascending aorta measured with an invasive flow probe as measured by 2D flow analysed with Medis [23].

Other reasons for differences between the vendors are probably due to various modes of vessel contouring during postprocessing. Placement of assessment planes is also prone to intraindividual differences since the plane positions of the reference 2D PC measurement were not transferred to the exact same position in the 4D flow postprocessing tools, especially in cases of aliasing, but were assessed in newly defined planes that were as comparable as possible to the reference 2D planes.

We found excellent correlation between systemic and pulmonary blood flow, and also between main versus summed branch pulmonary artery blood flow, which is important when establishing a new 4D flow software. An internal validation of systemic versus pulmonary flow volumes in patients without shunts, or of main versus combined branch pulmonary artery flow volumes, is recommended in 4D flow datasets [1]. The conservation of mass principle is also valid for 4D flow measurements, particularly when comparing results to 2D PC measurements as has been shown by Hanneman and coworkers [24]. In this study, 4D flow MRI resulted in accurate assessment of Qp:Qs ratios in the evaluation of intracardiac shunts, but it underestimated individual flow volumes. Other 4D versus 2D PC studies point out that scanner and sequence specific data validation has to be performed at each site, in particular with regards to phantom correction [25–27]. Net flow volumes compared between 4 and 2D PC measurements in the four major thoracic arteries agreed within limits of ± 15 ml per cycle in a previous study [26]. Another study achieved accurate flow quantification in the ascending aorta and pulmonary artery using PC-VIPR, a radially undersampled 4D flow sequence, with phantom correction compared with 2D PC measurements and cine SSFP sequences for ventricular volumetry [25].

Non-background phase corrected 4D flow data showed good correlation with 2D PC for each vessel separately, but internal consistency between vessels was poor for uncorrected 4D flow in our study. This might be due to opposing phase-offset errors in the different locations of the vessels. 4D flow analysis without phase-offset correction showed significant bias as compared to 2D PC for all software programmes in the order of 1.6–3.6 ml per heart beat (Table 1).

We found good to excellent interobserver reproducibility of 4D flow measurements for all four software programmes, which is important for application in daily routine in centres with more than one reader as well as for follow-up examinations in the same patient. Our aim was not to make comparisons between each software and the others, but to examine all software programmes for applicability for clinical use, in support of more global 4D flow adoption.

Given good diagnostic performance, processing time is interesting to consider for implementation into clinical routine. The cloud-based solution had the shortest processing time of the four software programmes examined on our computer systems, which may vary between centres.

Limitations of this study include its retrospective nature and the clinical heterogeneity of patients, which was necessary to achieve a sufficient sample size. For 2D PC sequences, the same velocity encoding was applied to all vessels and only increased in case of aliasing (Supplemental Table 1), in accordance with [28]. This could cause more noise in vessels with low flow velocities, however, our measurement of flow volumes is less susceptible to noise compared with, for instance, peak velocity measurements [29], as confirmed by good conservation of mass, used as an internal control measure. Our choice of processing software programms among the many available solutions was arbitrary, based on existing industry contacts and the willingness of the vendors to provide trial licenses. This does not imply any endorsement or discouragement of a specific vendor, and other processing tools should also be well tested before clinical application.

## Conclusion

The tested 4D flow postprocessing software yielded reproducible and valid net flow volumes of the aorta and pulmonary arteries. They all showed similar variation from 2D PC with acceptable percentage errors. Phase-offset correction as employed by each software minimised differences compared to no phase-offset correction and thus should always be implemented. There was good interobserver reproducibility for all software programmes. Thus, they can all be used in the clinical setting for flow assessments in patients with CHD.

### Supplementary Information

Below is the link to the electronic supplementary material.

Supplementary file1 (DOCX 15 KB)
