# Supplementary material for: Flow evaluation software for four-dimensional flow MRI: a reliability and validation study
Source: Radiol Med. 2023 Aug 24;128(10):1225–35. doi: 10.1007/s11547-023-01697-4 (PMC10547653; doi:10.1007/s11547-023-01697-4)
Supplement: Supplementary file 1 — Supplementary file1 (DOCX 15 KB) [file 11547_2023_1697_MOESM1_ESM.docx]

**Supplemental materials**

**Supplemental Table 1** Acquisition parameters of 2D PC and 4D flow in 47 cardiovascular MRI examinations

|  | 2D PC | 4D flow |
| --- | --- | --- |
| Flip angle | 20° | 15° |
| Repetition time; range | 6.3 – 6.6 ms | 4.1 – 4.6 ms |
| Spatial resolution; median (range) | 4 x 2.5(1.4–3.1) x 1.3(0.7–1.6) mm^3^ | 2.0 (1.6–2.4) mm^3^ |
| Repetition time; range | 6.3 – 6.6 ms | 4.1 – 4.6 ms |
| Echo time; range | 3.5 – 3.7 ms | 2.4 – 2.6 ms |
| Temporal resolution; median (range) | 52 (26 – 79) ms | 35 (18 – 69) ms |
| Velocity encoding; median (range) | 200 (200 – 350) cm/s | 160 (160 – 300) cm/s |

**Supplemental Table 2** Patient characteristics of 47 patients who underwent cardiovascular MRI with 2D PC and 4D flow MRI

| Age, median (IQR) | 15.7 (10.9 – 27.7) years |
| --- | --- |
| Male, frequency | 33/47 (70%) |
| Height, median (IQR) | 164 (138 – 174) cm |
| Weight, mean (SD) | 51 (25) kg |
| Heart rate, median (IQR) | 76 (69 – 85) bpm |
| Diagnosis / indication | n (%) |
| - Aortic pathology (bicuspid aortic valve, aortic coarctation) | 17 (36%) |
| - Tetralogy of Fallot, pulmonary valve atresia or stenosis, double outlet right ventricle | 10 (21%) |
| - Transposition of the great arteries, status post arterial switch | 3 (6%) |
| - Transposition of the great arteries, status post atrial switch | 3 (6%) |
| - Partial anomalous pulmonary venous connection, with shunt | 3 (6%) |
| - Total anomalous pulmonary venous connection, corrected | 1 (2%) |
| - Atrioventricular septal defect | 2 (4%) |
| - Tricuspid valve dysplasia | 1 (2%) |
| - Coronary aneurysms (Kawasaki disease) | 1 (2%) |
| - Ventricular tumour | 2 (4%) |
| - Rule out cardiomyopathy | 4 (8%) |

*IQR* interquartile range, *SD* standard deviation, *bpm* beats per minute;
